# Experimental and natural infections of severe acute respiratory syndrome-related coronavirus 2 in pets and wild and farm animals

**DOI:** 10.14202/vetworld.2022.565-589

**Published:** 2022-03-10

**Authors:** Gondo Mastutik, Ali Rohman, Reny I’tishom, Ignacio Ruiz-Arrondo, Ignacio de Blas

**Affiliations:** 1 Department of Anatomic Pathology, Faculty of Medicine, Universitas Airlangga, Surabaya 60131, Indonesia; 2 Department of Chemistry, Faculty of Science and Technology, Universitas Airlangga, Surabaya 60115, Indonesia; 3 Department of Medical Biology, Faculty of Medicine, Universitas Airlangga, Surabaya 60131, Indonesia; 4 Center for Rickettsioses and Arthropod-Borne Diseases, Hospital Universitario San Pedro–CIBIR, Logroño, Spain; 5 Department of Animal Pathology, Faculty of Veterinary Sciences, Instituto Universitario de Investigación Mixto Agroalimentario de Aragón (IA2), Universidad de Zaragoza, Spain

**Keywords:** animal disease, coronavirus disease 2019, infectious disease, pandemic, severe acute respiratory syndrome-related coronavirus 2

## Abstract

The severe acute respiratory syndrome-related coronavirus 2 (SARS-CoV-2) has spread globally and has led to extremely high mortality rates. In addition to infecting humans, this virus also has infected animals. Experimental studies and natural infections showed that dogs have a low susceptibility to SARS-CoV-2 infection, whereas domesticated cats and other animals in the family Felidae, such as lions, tigers, snow leopards, and cougars, have a high susceptibility to viral infections. In addition, wild white-tailed deer, gorillas, and otters have been found to be infected by SARS-CoV-2. Furry farm animals, such as minks, have a high susceptibility to SARS-CoV-2 infection. The virus appears to spread among minks and generate several new mutations, resulting in increased viral virulence. Furthermore, livestock animals, such as cattle, sheep, and pigs, were found to have low susceptibility to the virus, whereas chicken, ducks, turkeys, quail, and geese did not show susceptibility to SARS-CoV-2 infection. This knowledge can provide insights for the development of SARS-CoV-2 mitigation strategies in animals and humans. Therefore, this review focuses on experimental (both replication and transmission) *in vitro, ex vivo*, and *in vivo* studies of SARS-CoV-2 infections in pets and in wild and farm animals, and to provide details on the mechanism associated with natural infection.

## Introduction

In December 2019, a new human infectious respiratory disease outbreak was documented in Wuhan, Hubei Province, China [1]. The disease spread rapidly through human transmission and became a global pandemic. The disease had a high health impact, amounting to 422,510,872 cases and 5894,569 deaths by February19, 2022 [2]. The causative agent of the disease was identified as a new coronavirus strain [1]. As such, the disease was designated by the World Health Organization as the coronavirus disease 2019 (COVID-19), and the virus was named as the severe acute respiratory syndrome-related coronavirus 2 (SARS-CoV-2) by the International Committee on Taxonomy of Viruses [3]. The SARS-CoV-2 genome was 96.2% identical to the bat coronavirus RaTG13, *Rhinolophus affinis*, which was isolated at the Yunnan Province in China [4]. The increased genomic similarity and close phylogenetic tree prove that bats were the origin of SARS-CoV-2 [4]. The intermediate host appeared to be the Malayan pangolin (*Manis javanica*), whose genome Pangolin CoV is 91% identical to that of the SARS-CoV-2 and is 90.55% identical to that of the BatCoV RaTG13 [5]. Snakes and turtles can be considered as intermediate hosts, but this is still controversial and requires further investigation [6]. SARS-CoV-2 was transmitted to humans in Wuhan, China [1], and spread worldwide. The first cases of SARS-CoV-2 infections were identified in Australia on January 19, 2020 [7], in Europe on January 24, 2020 [8], in the Americas on February 29, 2020 [9], and in the African continent on March 5, 2020 [10].

SARS-CoV-2 belongs to the subgenus *Sarbecovirus* (genus *Betacoronavirus*) in the family *Coronaviridae*. It is an enveloped virus with a single-stranded, positive-sense ribonucleic acid (RNA) genome with a nucleotide size of ~30 kb [1,11]. The SARS-CoV-2 genome encodes four structural proteins: The nucleocapsid protein (N), membrane protein (M), envelope protein (E), and surface spike protein (S) [1,11]. The S-protein of SARS-CoV-2 is a glycosylated transmembrane protein that forms a homotrimer structure. It protrudes from the viral surface and mediates viral entry into host cells [12]. The S-protein of SARS-CoV-2 uses the angiotensin-converting enzyme 2 (ACE2) receptor as its binding receptor [13]. The sequence of the ­receptor-binding domain (RBD) of SARS-CoV-2, which includes the receptor-binding motif (RBM) of the S-protein, directly contacts the ACE2 receptor [13-15]. Human ACE2 is highly expressed in the lungs, heart, kidney, bladder, and gastrointestinal system [14,16]. ACE2 may also be present in mammalian cells. Analyses of the phylogenetic tree of animals that come into close contact with humans, such as pets and livestock, and ACE2 homology with the human ACE2 in various mammalian cells, showed a high degree of homology similarity [17-20]. *In silico* studies showed that ACE2 receptors from various domesticated animals, such as *Felis catus* (cat) and *Canis lupus familiaris* (dog), are highly homologous. *F. catus* and *C. lupus familiaris* have high degrees of similarities to human ACE2 of the orders of 85.2% and 83.4%, respectively [20]. Likewise, livestock, such as *Bos taurus* (cow), *Ovis aries* (sheep), and *Sus scrofa* *domesticus* (pig), exhibit high similarity [17-20]. The interactions between the ACE2 amino acids of the cat, dog, cow, sheep, and pig and the RBD and RBM of the SARS-CoV-2 S-protein were predicted to allow the binding of SARS-CoV-2 [17,18]. Analyses of changes in the binding energy (DDG) of the SARS-CoV-2 S-protein and the ACE2 complexes from cats, dogs, cows, sheep, and pigs showed that these animals belong to the risk category of SARS-CoV-2 infections, as indicated by DDG values ≤3.72 [21]. Consequently, these findings support the susceptibility of domesticated and livestock animals to SARS-CoV-2 infections.

In addition to infecting humans, SARS-CoV-2 has been reported to infect animals. Experimental infections of SARS-CoV-2 in animals have been reported in cats, dogs, ferrets, and poultry (March 2020) [22]. SARS-CoV-2 RNA has also been detected by the reverse transcription-polymerase chain reaction (RT-PCR) in pets from owners with confirmed COVID-19 infections. The first case was reported in dogs in Hong Kong (February 2020) [23], and in cats in Hong Kong (February-August 2020) [24], Belgium (March 2020) [25], and France (April 2020) [26]. The serological surveys found antibodies against SARS-CoV-2 in cats from Wuhan, China (during January-March 2020) [27] and in cats and dogs in Italy (May 2020) [28]. Furthermore, SARS-CoV-2 was detected in wild animals, such as lions, and tigers at the Bronx Zoo in New York City, United States of America (USA) in March 2020 [29,30]. Recently, antibodies to SARS-CoV-2 were also detected in wild white-tailed deer (*Odocoileus virginianus*) during January-March 2021 in four states in the USA [31]. SARS-CoV-2 RNA was detected in wastewater in Australia (published online on April 18, 2020) [32] and in the USA in January 2021 [33]. Both the SARS-CoV-2 RNA virus and antibodies against SARS-CoV-2 were also detected in farmed minks. The first case was also detected in the Netherlands during April and May 2020 [34]. Furthermore, SARS-CoV-2 was reported to be transmitted from humans to minks, which led to the development of zoonotic diseases that have been proved to be transmitted back to humans [35]. Many animals, including those with experimentally induced or natural infections, are not yet known for their susceptibility to SARS-CoV-2 infections and many cases of natural infection have not been reported.

Therefore, this review focuses on experimental studies of SARS-CoV-2 infections, including *in vitro*, *ex vivo*, and *in vivo* studies on viral replication and transmission capabilities in pets and wild and farm animals. This explains the evidence of natural cases of SARS-CoV-2 infections in domesticated animals, including cats, dogs, minks, and wild animals, such as big cats and wild deer, in all continents until October 2021. This knowledge can be used to determine policy strategies adopted to mitigate the spread of infectious diseases in both animals and humans.

## SARS-CoV-2 Infections in Pets

### SARS-CoV-2 infections in cats

**Figure-1:**
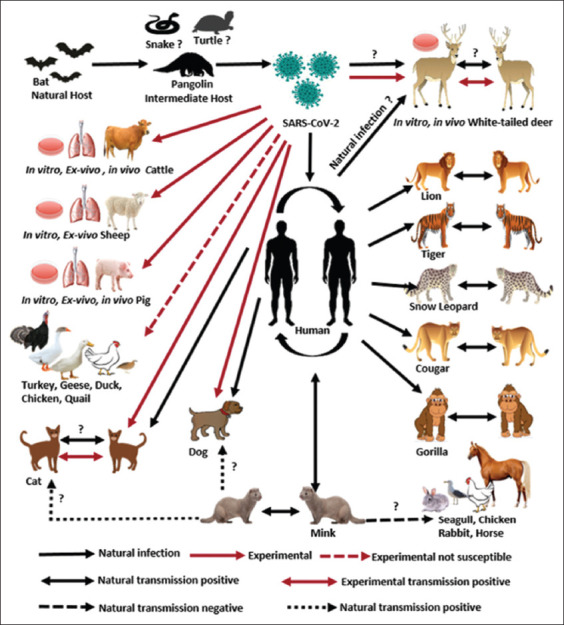
Experimental and natural infections of the severe acute respiratory syndrome-related coronavirus 2 (SARS-CoV-2) in pets and wild and farm animals [4,5,23-29,31,34,35,40,41,43-66,91]. SARS-CoV-2 was assumed to originate in the bat species [4], and the virus was then transmitted from them to humans through an intermediate animal host, that is, pangolins [5]. Indeed, the spread of this virus among humans and many animals has been reported widely. These animals include domestic cats [23-28,51-55], dogs [23,26,28,52,54,55], and wild Felidae families, such as tigers [29,65,66], lions [29,62-64], snow leopards [60,61] and cougars [59], as well as gorilla [91]. It was confirmed that the animals acquired viral infection from humans infected with SARS-CoV-2. The virus spread among these group animals in the same cage. Another wild animal susceptible to SARS-CoV-2 infection is the white-tailed deer [31]. Experimentally [43,44], SARS-CoV-2 has been shown to replicate *in vitro* and transmit *in vivo* among these animals and vertically to the fetus. In natural infections, white-tailed deer were found positive for the SARS-CoV-2 infection and had high seroprevalence [31], although the source of transmission from human or nature is still unclear. Minks were naturally infected with SARS-CoV-2 from humans, and subsequently spread the virus among them, and the virus was transmitted back to humans [34,35,56-58]. It is not clear whether minks can transmit the virus to other animals, such as dogs, cats, seagulls, chickens, horses, and rabbits in farms. Experimentally, SARS-CoV-2 cannot infect poultries, such as chickens, ducks, geese, turkeys, and quails [45,50]. The virus was reported to infect several livestock animals experimentally, including cattle [40,41], sheep [41], and pigs [22,41,45-49], but natural infections have not been reported.

Some animals have been known to be experimentally infected with the SARS-CoV-2 virus. In addition, there has been evidence of natural infections in various animals from several countries, including China, which was the first country in which human infections were found, and in other countries in Asia, Europe, Australia, Africa, and the Americas. Some studies conducted to challenge animals against SARS-CoV-2 infection are presented in Table-1 [22,36-50], whereas natural infections in animals, including domestic animals, farm animals, and wild animals, are listed in Table-2 [23-29,31,34,35,51-66], and natural infections in the USA are listed in Table-3 [67-90]. Experimental infections and natural cases with the presumed sources of infection and their transmission are summarized in Figure-1 [4,5,23-29,31,34,35,40,41,43-66,91].

**Table-1:** Experimental SARS-CoV-2 infection in animals.

Species	Method	Age	Route and Dose	Virus Isolation	Clinical Sign	Replication virus	Antibody to SARS-CoV-2	Transmission	Susceptibility	Reference
Cat (*Felis catus*)	*In vivo*	70-100 days	Intranasal with 10^5^ PFU of CTan-H	SARS-CoV-2/CTan/human/2020/Wuhan (CTan-H)	N/A	Yes, and shed virus	Yes	Yes	High	[22]
	*In vivo*	5-18-week-old	Intranasal, oral, intratracheal, ocular by 5.2×10^5^ PFU	UT-NCGM02/Human/2020/Tokyo	No	Yes, and shed virus	Yes	Yes	High	[36]
	*In vivo*	6-9 months	Intranasal with 10^5^ PFU of CTan-H	SARS-CoV-2/CTan/human/2020/Wuhan (CTan-H)	N/A	Yes, and shed virus	Yes	Yes	High	[22]
	*In vivo*	5-8 years	Nares (500 mL/nare) for a total volume of 1 mL (3.0×10^5^ PFU)	SARS-CoV-2 virus strainWA1/2020WY96	No	Yes, and shed virus	Yes	Yes	High	[37]
	*In vivo*	15-18-week-old)	Intranasal, oral, intratracheal, ocular by 5.2×10^5^ PFU	UT-NCGM02/Human/2020/Tokyo	No	Yes, and shed virus	Yes	Yes	High	[38]
	*In vivo*	4.5-5 months	Intranasal and oral with 1×10^6^ TCID^50^/mL	SARS-CoV-2 USA-WA1/2020 strain	No	Yes, and shed virus	Yes	Yes	High	[39]
Dog (*Canis lupus*)	*In vivo*	3 months	Intranasal with 10^5^ PFU of CTan-H	SARS-CoV-2/CTan/human/2020/Wuhan (CTan-H)	N/A	Yes, but not shed virus	Yes	No	Low	[22]
	*In vivo*	5-6 years	Nares (500 mL/nare) for a total volume of 1 mL (1.4×10^5^ PFU)	SARS-CoV-2 virus strainWA1/2020WY96	No	Yes, but not shed virus	Yes	N/A	Low	[37]
Cattle (*Bos taurus*)	*In vitro*: bovine turbinate, *Bos taurus* trachea normal (EBTr (NBL-4)), cow pulmonary artery epithelial, primary fetal bovine lung, and fetal bovine kidney cells	N/A	Multiplicity of infection of 1 or 0.1 (MOI=1 or 0.1)	SARSCoV-2 isolate TGR/NY/20	N/A	Not replicate	N/A	N/A	N/A	[40]
Cattle (*Bos taurus*)	*Ex vivo*: Respiratory *ex vivo* organ cultures	18 months	Infected with 10^3^ TCID_50_/mL	SARS-CoV-2/INMI1-Isolate/2020/Italy (D614); SARS-CoV-2/IZSAM/46419 (D614G)	N/A	Yes	N/A	N/A	N/A	[41]
	*In vivo*	6 weeks	Intratracheal or intravenous, 5 mL each respective route	SARSCoV-2 isolate TGR/NY/20	High temp and mild caught	Yes, but not shed virus	Yes	N/A	Low	[40]
	*In vivo*	<1 year	Intranasal with 1×10^5^ 50% tissue culture infectious dose of SARS-CoV-2	SARS-CoV-2 Strain 2019_nCoV Muc-IMB-1	N/A	Yes, but not shed virus	Yes	No	Low	[42]
Sheep (*Ovis aries*)	*Ex vivo*: Respiratory *ex vivo* organ cultures	10 months	Infected with 10^3^ TCID_50_/mL	SARS-CoV-2/INMI1-Isolate/2020/Italy (D614); SARS-CoV-2/IZSAM/46419 (D614G)	N/A	Yes	N/A	N/A	Low	[41]
White tail deer (*Odocoileus virginianus*)	*In vitro*: Deer lung cells	N/A	Inoculated multiplicities of infection of 0.1 and 1	SARS-CoV-2 isolate TGR/NY/20	N/A	Yes	N/A	N/A	N/A	[43]
	*In vitro*: lung cells isolated from white-tailed deer, mule deer and elk	N/A	Infected at approximately 0.1 MOI	SARS-CoV-2 lineage A WA1 strain	N/A	Yes, in white-tailed deer, mule deer lung cells	N/A	N/A	N/A	[44]
	*In vivo*	6 weeks	Intranasal with 5 mL (2.5 mL per nostril) of a virus suspension containing 10^6.3^ TCID_50_/mL	SARS-CoV-2 isolate TGR/NY/20	Subclinical viral infection	Yes, and shed virus	Yes	Yes	High	[43]
	*In vivo*	2 years	Intranasal and oral with 2 mL dose of 1×10^6^ TCID_50_ per animal	1:1 titer ratio of lineage A WA1 and the alpha VOC B.1.1.7 strain	Subclinical viral infection	Yes, and shed virus	Yes	Yes, and vertical	High	[44]
Pig (*Sus scrofa domesticus*)	*In vitro*: Porcine kidney-15, swine kidney -6, and swine testicle	N/A	Inoculated with 10^5^ TCID_50_ SARS-CoV-2	SARS-CoV-2 2019_nCoV Muc-IMB-1	N/A	Yes, in SK-6 and ST	N/A	N/A	N/A	[45]
	*In vitro*: ST and PK-15 cell lines	N/A	0.05 MOI of passage 3 of the VeroE6-passaged SARS-CoV-2	SARS-CoV-2 USA-WA1/2020 isolate	N/A	Yes, in ST and PK-15	N/A	N/A	N/A	[46]
	*Ex vivo*: Respiratory *ex vivo* organ cultures	12 months	Infected with 10^3^ TCID_50_/mL	SARS-CoV-2/INMI1-Isolate/2020/Italy (D614); SARS-CoV-2/IZSAM/46419 (D614G)	N/A	Not detected	N/A	N/A	N/A	[41]
	*In vivo*	5 weeks	Oral, intranasal, intratracheal with 1×10^6^ TCID_50_ of SARSCoV-2	SARS-CoV-2 USA-WA1/2020 isolate	No	Not detected	Not detected	No	No	[46]
	*In vivo*	N/A	Intranasal with 10^5^ PFU of CTan-H	SARS-CoV-2/CTan/human/2020/Wuhan (CTan-H)	N/A	Not detected	Not detected	No	No	[22]
	*In vivo*	9 weeks	Intranasal with 10^5^ TCID_50_ SARS-CoV-2	SARS-CoV-2 2019_nCoV Muc-IMB-1	No	Not detected	Not detected	N/A	No	[45]
	*In vivo*	5-6 weeks	Intranasal, intratracheal, intramuscular and intravenous 10^5.8^ TCID_50_	SARS-CoV-2 isolate (GISAID ID EPI_ISL_510689)	No	Yes, but not shed virus	Yes, at IM, IV route	N/A	No	[47]
	*In vivo*	8 weeks	Intranasal and pharynx routes of 10^6^ PFU/animal	SARS-CoV-2 isolate hCoV-19/Canada/ON-VIDO-01/2020	No, but an animal yes)	Yes, but not shed virus		No	Low	[48]
	*In vivo*	3 weeks	Intravenous, intratracheal, and intranasal 6.8×10^6^ TCID_50_/mL	SARS-CoV-2 isolate used in our study (TGR1/NY/20)	No	Yes, but not shed virus	Yes, but not sustained	No	Low	[49]
Chickens (*Gallus gallus domesticus*)	*In vivo*: Embryonating chicken eggs	N/A	Yolk sac, chorioallantoic sac, and chorioallantoic membrane	USA-WA1/2020 isolate of SARS-CoV-2 (BEI NR-58221)	N/A	Not detected	Not detected	N/A	No	[50]
	*In vivo*: ECE	N/A	Inoculated SARS-CoV-2 in ECE	SARS-CoV-2 2019_nCoV Muc-IMB-1	N/A	Not detected	N/A	N/A	No	[45]
	*In vivo*	5 weeks	Intranasal with 10^5^ TCID_50_ SARS-CoV-2	SARS-CoV-2 2019_nCoV Muc-IMB-1	No	Not detected	Not detected	N/A	No	[45]
	*In vivo*	N/A	Challenged with SARS-CoV-2	USA-WA1/2020 isolate of SARS-CoV-2 (BEI NR-58221)	No	Not detected	Not detected	N/A	No	[50]
	*In vivo*	N/A	Intranasal with 10^5^ PFU of CTan-H	SARS-CoV-2/CTan/human/2020/Wuhan (CTan-H)	N/A	Not detected	Not detected	No	No	[22]
Turkeys *(Meleagris gallopavo*)	*In vivo*	N/A	Challenged with SARS-CoV-2	USA-WA1/2020 isolate of SARS-CoV-2 (BEI NR-58221)	No	Not detected	Not detected	N/A	No	[50]
Ducks (*Anas platyrhynchos domesticus*)	*In vivo*	N/A	Intranasal with 10^5^ PFU of CTan-H	SARS-CoV-2/CTan/human/2020/Wuhan (CTan-H)	N/A	Not detected	Not detected	No	No	[22]
	*In vivo*	N/A	Challenged with SARS-CoV-2	USA-WA1/2020 isolate of SARS-CoV-2 (BEI NR-58221)	No	Not detected	Not detected	N/A	No	[50]
Quail (*Coturnix japonica*)	*In vivo*	N/A	Challenged with SARS-CoV-2	USA-WA1/2020 isolate of SARS-CoV-2 (BEI NR-58221)	No	Not detected	Not detected	N/A	No	[50]
Geese (*Anser cygnoides*)	*In vivo*	N/A	Challenged with SARS-CoV-2	USA-WA1/2020 isolate of SARS-CoV-2 (BEI NR-58221)	No	Not detected	Not detected	N/A	No	[50]

PFU=Plaque-forming units, SARS-CoV-2=Severe acute respiratory syndrome-related coronavirus 2, N/A=Not available

**Table-2:** Natural infections of SARS-CoV-2 in pet, wild and farm animals.

Species	Location	Sample Sources	Total sample	Total Positive	Clinical Sign	RNA Virus Detected	Antibody to SARS-CoV-2	Reference
Cat (*Felis catus*)	Wuhan (China)	Animal shelters, pet hospital, and Households confirmed COVID-19	102	15	N/A	Negative	Positive	[27]
	Hong Kong (China)	Households confirmed COVID-19	7	0	Asymptomatic	Negative	Negative	[23]
	Hong Kong (China)	Households confirmed COVID-19	50	6	Asymptomatic	Positive	Positive	[24]
	Spain	Households confirmed COVID-19	8	1	Asymptomatic	Positive	N/A	[52]
	Spain	Households confirmed COVID-19	1	1	Feline hypertrophic cardiomyopathy, but the animal was also infected by SARS-CoV-2	Positive	Positive	[53]
	Belgium	Households confirmed COVID-19	1	1	Mild gastrointestinal and respiratory signs	Positive	Positive	[25]
	France	Households confirmed COVID-19	22	1	Mild respiratory and digestive signs.	Positive	Positive	[26]
	Italy	Households confirmed COVID-19 or living in geographic areas that were severely affected by COVID-19	191	11	Not clearly explained	Negative	Positive	[28]
	Rio de Janeiro (Brazil)	Households confirmed or not confirmed COVID-19 and stray animals	49	1	N/A	Negative	Positive	[54]
	Rio de Janeiro (Brazil)	Households confirmed COVID-19	10	4	Unspecified, mild, reversible signs, respiratory or gastrointestinal signs	Positive	Positive	[55]
	New York (USA)	Households confirmed COVID-19	2	2	Sneezing, clear ocular discharge, and mild lethargy	Positive	N/A	[51]
Tiger (*Panthera tigris*)	New York (USA)	Bronx Zoo	5	4	Mild respiratory signs	Positive	N/A	[29]
	Jakarta (Indonesia)	Ragunan Jakarta Zoo	2	2	Mild respiratory signs and general symptoms	Positive	N/A	[65,66]
Lion (*Panthera leo*)	New York (USA)	Bronx Zoo	3	3	Mild respiratory signs	Positive	N/A	[29]
	Catalonia (Spain)	Barcelona Zoo	12	3	Mild respiratory signs	Positive	Positive	[64]
	Tamil Nadu (India)	Arignar Anna Zoological Park in Chennai	11	9	Mild respiratory signs and general symptoms	Positive	N/A	[62]
	Uttar Pradesh and Rajasthan (India)	Lion Safari Park, Etawah and Nahargarh Biological Park	3	12	Mild respiratory signs and general symptoms	Positive	Positive	[63]
Snow leopard (*Panthera uncia*)	Louisville (USA)	Louisville Zoo	3	3	Mild respiratory signs	Positive	N/A	[61]
	San Diego (USA)	San Diego Zoo	1	1	N/A	Positive	N/A	[60]
Cougar (*Puma concolor*)	Texas (USA)	Texas animals	1	1	Mild respiratory signs	Positive	N/A	[59]
Dog (*Canis lupus familiaris*)	Hong Kong (China)	Quarantine animal from households with confirmed COVID-19	15	2	Asymptomatic	Positive	Positive	[23]
	Spain	Households confirmed COVID-19	12	0	Asymptomatic	Negative	N/A	[52]
	France	Households confirmed COVID-19	11	0	Mild respiratory and digestive signs	Negative	Negative	[26]
	Italy	Households confirmed COVID-19 or living in geographic areas that were severely affected by COVID-19	451	15	Not clearly explained	Negative	Positive	[28]
	Rio de Janeiro (Brazil)	Households confirmed or not confirmed COVID-19 and stray animals	47	1	N/A	Negative	Positive	[54]
	Rio de Janeiro (Brazil)	Households confirmed COVID-19	29	9	Unspecified, mild, reversible signs, respiratory or gastrointestinal signs	Positive	Positive	[55]
White tail deer (*Odocoileus virginianus*)	Michigan, Pennsylvania, Illinois, New York (USA)	Wild white-tailed deer population	385	152	N/A	N/A	Positive	[31]
Mink (*Neovison vison*)	The Netherlands	Mink farms	16 mink farms	N/A	Mild to severe respiratory distress	Positive	N/A	[34,35,56]
	Denmark	Mink farms	1147 mink farms	290 mink farms	N/A	Positive	N/A	[57]
	Poland	Mink farms	28 mink farms	1 mink farm	N/A	Positive (70% sample)	Positive (30% sample)	[58]
Guinea pig (*Cavia porcellus)*	Spain	Households confirmed COVID-19	1	1	Asymptomatic	Negative	N/A	[52]
Rabbit (*Oryctolagus cuniculus*)	Spain	Households confirmed COVID-19	1	2	Asymptomatic	Negative	N/A	[52]

SARS-CoV-2=Severe acute respiratory syndrome-related coronavirus 2, N/A=Not available

**Table-3:** Natural infection of severe acute respiratory syndrome-related coronavirus 2 in USA reported by OIE.

Species	No. of follow-up report	Location	Date of outbreak	Suspect	Case	Death	Clinical signs	Reference
Domestic cat (*Felis catus*)	No. 2 and 3	Nassau County, Nassau, New York,	April 1, 2020	1	1	-	Respiratory signs	[67,68]
	No. 2 and 3	Orange County, Orange, New York	April 6, 2020	2	1	-	Respiratory signs	[67,68]
	No. 5	Carver County, Carver, Minnesota	May 20, 2020	1	1	-	Respiratory signs	[75]
	No. 6 and 7	Cook County, Cook, Illinois	May 19, 2020	1	1	-	Respiratory signs	[76,77]
	No. 9	Orange County, Orange, California	June 26, 2020	1	1	1	Respiratory and cardiac signs	[78]
	No. 9	Orange County, Orange, California	June 27, 2020	1	1	-	Asymptomatic	[78]
	No. 11	Brazos County, Brazos, Texas	June 28, 2020	1	1	-	Asymptomatic	[79]
	No. 11	Maricopa County, Maricopa, Arizona	July 10, 2020	1	-	-	N/A	[79]
	No. 12	Brazos County, Brazos, Texas	July 17, 2020	1	1	-	Asymptomatic	[80]
	No. 14	Brazos County, Brazos, Texas	July 29, 2020	3	1	-	Asymptomatic	[81]
	No. 16	Coweta County, Coweta, Georgia	July 14, 2020	1	1	-	Respiratory signs	[82]
	No. 16	Hartford County, Hartford, Maryland	August 10, 2020	5	1	-	Respiratory signs	[82]
	No. 16	Contra Costa County, Contra Costa, California	August 13, 2020	1	1	-	Respiratory signs	[82]
	No. 17	Rapides Parish, Rapides, Louisiana	August 17, 2020	4	1	-	Respiratory signs	[69]
	No. 18	Brazos County, Brazos, Texas	August 11, 2020	1	1	-	Asymptomatic	[70]
	No. 18	Somervell County, Somervell, Texas	August 12, 2020	9	1	-	Asymptomatic	[70]
	No. 18	Brazos County, Brazos, Texas	August 21, 2020	1	1	-	Asymptomatic	[70]
	No. 19	Fayette County, Fayette, Kentucky	September 6, 2020	3	1	-	Respiratory signs	[71]
	No. 20	Brazos County, Brazos, Texas	September 11, 2020	1	1	-	Asymptomatic	[72]
	No. 21	Lee County, Lee, Alabama	September 25, 2020	4	2	1	Respiratory signs	[73]
	No. 23	Cumberland County, Cumberland, Pennsylvania	October 02, 2020	1	1	-	Respiratory signs	[74]
Total of Domestic cat (*Felis catus*)				44	21	2		
Domestic dogs (*Canis lupus familiaris*)	No. 4	Richmond County, Richmond, New York	April 15, 2020	2	1	-	Respiratory signs	[86]
	No. 8	Berrien County, Berrien, Georgia	June 22, 2020	3	1	-	Neurological signs	[83]
	No. 9	Orange County, Orange, California	June 28, 2020	1	1	-	Asymptomatic	[78]
	No. 10	Charleston County, Charleston, South Carolina	June 26, 2020	3	1	-	Respiratory signs	[84]
	No. 11	Brazos County, Brazos, Texas	June 28, 2020	2	-	-	Asymptomatic	[79]
	No. 11	Maricopa County, Maricopa, Arizona	July 10, 2020	3	1	-	Respiratory signs	[79]
	No. 12	Brazos County, Brazos, Texas	July 17, 2020	2	-	-	N/A	[80]
	No. 13	Livingston Parish, Livingston, Louisian	July 22, 2020	2	1	-	N/A	[85]
	No. 14	Brazos County, Brazos, Texas	July 28, 2020	1	1	-	Asymptomatic	[81]
	No. 14	Moore County, Moore, North Carolina	August 4, 2020	2	1	1	Respiratory signs and cardiac arrest	[81]
	No. 16	Hartford County, Hartford, Maryland	August 10, 2020	1	-	-	N/A	[82]
	No. 17	Rapides Parish, Rapides, Louisiana	August 17, 2020	1	-	-	N/A	[69]
	No. 18	Brazos County, Brazos, Texas	August 11, 2020	1	1	-	Respiratory signs	[70]
	No. 18	Brazos County, Brazos, Texas	August 12, 2020	2	1	-	Respiratory signs	[70]
	No. 18	Somervell County, Somervell, Texas	August 12, 2020	2	-	-	Asymptomatic	[70]
	No. 18	Brazos County, Brazos, Texas	August 21, 2020	1	-	-	N/A	[70]
	No. 18	Brazos County, Brazos, Texas	August 21, 2020	1	1	-	Asymptomatic	[70]
	No. 20	Brazos County, Brazos, Texas	September 14, 2020	1	1	-	Respiratory signs	[72]
	No. 23	Brazos County, Brazos, Texas	October 01, 2020	2	1	-	Respiratory signs	[74]
Total of Domestic dogs (*Canis lupus familiaris*)				33	13	1		
Domestic American Mink (*Neovison vison*)	No. 15	Utah, Utah	June 26, 2020	20,000	N/A	3,524	Respiratory signs and death	[87]
	No. 15	Utah, Utah	August 2, 2020	8,983	N/A	1,451	Respiratory signs and death	[87]
	No. 16	Utah, Utah	August 03, 2020	6,326	N/A	1,554	Respiratory signs and death	[82]
	No. 16	Utah, Utah	August 05, 2020	3,643	N/A	1,119	Respiratory signs and death	[82]
	No. 16	Utah, Utah	August 05, 2020	1,705	N/A	205	Respiratory signs and death	[82]
	No. 19	Utah, Utah	September 08, 2020	1,500	N/A	59	Respiratory signs and death	[71]
	No. 20	Utah, Utah	September 07, 2020	600	N/A	146	Respiratory signs and death	[72]
	No. 20	Utah, Utah	September 20, 2020	14,000	N/A	247	Respiratory signs and death	[72]
	No. 21	Michigan, Michigan	September 27, 2020	17,000	N/A	2,000	Respiratory signs and death	[73]
	No. 21	Wisconsin, Wisconsin	September 30, 2020	14,600	N/A	1,800	Respiratoandry signs and death	[73]
	No. 22	Utah, Utah	September 29, 2020	300	N/A	126	Respiratory signs and death	[88]
	No. 25	Utah, Utah	October 08, 2020	3,000	N/A	373	Respiratory signs and death	[89]
	No. 25	Wisconsin, Wisconsin	October 19, 2020	22,500	N/A	2,200	Respiratory signs and death	[89]
	No. 25	Utah, Utah	October 22, 2020	13,200	N/A	585	Respiratory signs and death	[89]
	No. 25	Utah, Utah	October 25, 2020	38,000	N/A	739	Respiratory signs and death	[89]
	No. 26	Oregon, Oregon	October 22, 2020	12,000	N/A	2	Respiratory signs and death	[90]
Total of Domestic American Mink (*Neovison vison*)				177,357		16,130		

N/A=Not available

Experimental studies on SARS-CoV-2 replication and transmission have been observed in cats [22,36-39]. The viral replication was investigated in juvenile [22], sub-adult [22,36,38,39], and adult cats [37]. In juvenile cats, SARS-CoV-2 was efficiently replicated in the upper and lower respiratory tracts [22]. In young cats, viral RNA was replicated and detected in nasal or oropharyngeal swabs during the 1^st^ week post-infection and peak viral shedding at 4-5 days post-infection [36,38,39]. In sub-adult cats, the virus replicated efficiently in the upper respiratory tract in the beginning of infection, but some replicated in the lower respiratory tract and in the small intestine [22]. Viral replication and shed viruses were also found orally and nasally up to days 5 post-infection in adult cats [37].

All young and sub-adult cats did not show clinical signs and symptoms of the disease [36,38,39]. However, the histopathological features of the respiratory tract showed lymphocytic inflammation during early infection in combination with mixed inflammation during the peak infection period and decreased during the recovery period [38]. Moderate lesions were found in the lungs in the early infection stage [38,39] but tended to persist during the clearance of the virus, during which the lesions progressed to chronic histopathological features [38]. Adult cats exhibited no clinical signs of diseases, but histopathological features indicated subclinical pathological changes in the upper respiratory tract [37]. Juvenile cats exhibited massive lesions in the upper and lower respiratory tracts, suggesting that young cats are more susceptible to SARS-CoV-2 infections than adult cats [22]. Viral RNA obtained from nasal swabs was not detectable in re-infected animals. Microscopically, the lungs appeared with peribronchial fibrosis and thickening of the alveolar septa [38]. All these experiments revealed that cats were highly susceptible to SARS-CoV-2 infection. The virus can replicate efficiently in the respiratory tract and then shed nasally and orally, even though the cats did not exhibit any clinical symptoms [22,36-39].

The transmission of SARS-CoV-2 from inoculated cats to naive-contact cats was observed in juvenile, sub-adult, and adult cats [22,36-39]. In naive co-housed cats, viral RNA was detected in rectal swabs and in the upper respiratory tract tissues at days 1-3 post-exposure, persisted at days 5-9 post-exposure, and the shed virus reached the peak at days 4-5 post-exposure [22,36,37,39]. Viral RNA in the naive co-housed cats was detected in the upper respiratory tract and esophagus but not in the lung or other organs on day 5 post-exposure [37]. The virus was optimally replicated and longer in the upper respiratory tract [36-39] than in the lower respiratory tract [39]. Subsequently, the virus was excreted and spread from the oral or nasal cavity [36,37,39] with respiratory droplets to the naive co-housed cats through the airborne route [22]. This suggested that cats allowed viral replication and the virus were then transmitted by direct contact (co-housed) to naive cats. It is proved the transmission of SARS-CoV-2 from infected cats to other cats [22,37,39].

In addition, re-challenges of SARS-CoV-2 infections in cats were observed at 21 days [39] and 28 days after the first infection [38]. A re-challenge at 21 days showed that the animals were asymptomatic, but viral RNA was found high in the upper respiratory tract and gastrointestinal tissue, and low in the lower respiratory tract, lymphatic tissues, heart, and olfactory bulb [39]. On the contrary, re-infection at 28 days showed no viral RNA detection in nasal, oral, and rectal swabs or in the respiratory tract, brain, liver, spleen, kidney, small and large intestines, heart, and eyelid tissues on day 3 after re-infection [38]. This may be related to the immunity to SARS-CoV-2. Immunoglobulin M bound to the RBD of SARS-CoV-2 was detected on day 7 and reached the peak on day 14, and decreased up to day 28, whereas immunoglobulin G was detected on day 7 post-infection and continued to increase up to day 28; it then reached a plateau on day 42 post-infection [37]. Immunity on day 28 after the first infection may have reached its peak to provide the protective effect on the second challenge infection [37].

In addition to the proof on experimentally induced SARS-CoV-2 infections, some studies reported natural infections in several animals, as summarized in Table-2. In Hong Kong, the natural infection with SARS-CoV-2 has been observed in 6 of 50 (12%) quarantined animals from households or animals with close contact with patients with COVID-19 [24]. A serological study in cats collected from animal shelters, pet hospitals, and households with COVID-19 in Wuhan, China, from January to March 2020 showed that 15 of 102 (14.7%) cats were positive for antibodies against SARS-CoV-2. However, all nasopharyngeal and anal swabs were negative for SARS-CoV-2 viral RNA [27]. In Thailand, a serological survey was conducted on cats from April to December 2020 and showed that 4 of 1112 sera antibodies were positive to antibodies against SARS-CoV-2 [92].

Natural SARS-CoV-2 infection was reported in Europe, including Belgium, Spain, France, and Italy. In Belgium, a cat from the owner with COVID-19 in March 2020 was positive for the SARS-COV-2 viral RNA and developed neutralizing antibodies against SARS-CoV-2 [25]. In La Rioja, Northern Spain, a study on 23 asymptomatic animals in quarantine from April 8 to May 4, 2020, including eight cats from an owner with COVID-19, found that one of eight cats was positive for SARS-CoV-2 viral RNA based on RT-PCR [52]. Two cats of the owners who died from COVID-19 on March 18, 2020, in Spain, was reported seroconverted to SARS-CoV-2; however, viral RNA was detected in the first cat but not in the second cat [53]. In France, a cohort study conducted on 22 cats from owners who were infected, or suspected to be infected, showed that a cat was positive for viral RNA and antibodies. This cat had mild respiratory and digestive signs. Furthermore, the genomic analysis of SARS-CoV-2 from this cat revealed a genome resembling the SARS-CoV-2 genome in most French humans [26]. In addition, another study in France reported that seroprevalent antibodies against SARS-CoV-2 were increased in cats and dogs from the confirmed COVID-19 household cases by 21.3% and by 2.6% in no confirmed COVID-19 households [93]. In Italy, an epidemiological study involving 277 cats living in SARS-CoV-2-positive households or in the geographic areas severely affected by COVID-19 found that several animals developed neutralizing antibodies. In contrast, viral RNA was negative in all swab samples [28].

SARS-CoV-2 infections in cats were reported in Rio de Janeiro, Brazil. Data were collected from June to August 2020 from cats living in a household with owners with confirmed COVID-19 and stray animals. Interestingly, serum from a stray cat tested positive for antibodies to SARS-CoV-2, even though the tests were negative for viral RNA [54]. Another study in the same city showed that cats from households with owners positive for COVID-19 showed positive results for viral RNA (3 of 10 household cats) and developed a neutralizing antibody to SARS-CoV-2 (two of four cats) [55].

The first infection with SARS-CoV-2 in cats in the USA was reported in April 2020 [67,68]. The other cases were reported by the World Organization for Animal Health (OIE) in the follow-up reports, with numbers of 2, 3, 5, 6, 7, 9, 11, 12, 14, 16, 17, 18, 19, 20, 21, and 23 [67-82], as listed in Table-3. SARS-CoV-2 infections were confirmed by RT-PCR in a total of 44 suspected cats and 21 cats [67-82]. In the first case, two cats had clinical signs of respiratory illness from owners with COVID-19. Both cats were positive for SARS-CoV-2 RNA and developed antibodies against SARS-CoV-2 [51,67]. Recently, in Texas, USA, infection with SARS-CoV-2 was reported in cats of the COVID-19 household, which showed 17.6% of the cats were positive for SARS-CoV-2, and 43.8% of the cats were found to have neutralizing antibodies against SARS-CoV-2 [94].

The susceptibility of animals to SARS-CoV-2 infection was predicted by comparing ACE2 of animals and humans [17,18,95]. ACE2 is the receptor that interacts with the spike protein of SARS-CoV-2 that allows viral entry to host cells [17,18,95]. Cats ACE2 presented four amino acid changes related to Gln24Leu, Asp30Glu, Asp38Glu, and Met82Thr [95]. The residue Asp30 in ACE2 was negatively charged and formed a salt bridge with Lys417 (positively charged) in the S-protein of SARS-CoV-2. This stable bridge is located in the middle of the surface interaction [95]. The Asp30 to Glu mutation residue formed more stable bridges than Asp30 residue [95]. His34, located in the center of surface interaction, and the N-glycosylation site at residue Asn90 were similar to those of human ACE2 [17,18,95]. This predicted that cat ACE2 was suitable as the attachment site of the S-protein of SARS-CoV-2 [17,18,95]. The findings of these *in silico* studies were consistent with experimental studies [22,36-39] and with naturally infected cases of SARS-CoV-2 in cats [24,25,52,53,94]. This may also explain the susceptibility of cats to SARS-CoV-2 infection [24,25,52,53,94], and the ability of the virus to replicate and transmit between cats [22,36,37].

SARS-CoV-2 infections in *in vivo* studies [22,36-39], and mainly in naturally infected cases, did not result in clinical symptoms [96]. Although asymptomatic, thickening of the alveolar septa was found histopathologically, which indicated chronic lung inflammation [38]. Recently, an unusual clinical manifestation has been documented, which included severe myocarditis and impaired general health in cats infected by the B.1.1.7 variant of SARS-CoV-2 [97]. It was also reported previously in human patients that symptoms of acute myocarditis developed in more than 25% of critical cases because of SARS-CoV-2 infections [14]. A systematic review reported that cats developed variable mild to severe respiratory signs, with predominant presentations of sneezing and coughing, gastroenteritis (vomit and diarrhea), diminishing general health status (fever, lethargy, and lack of appetite), cardiovascular signs (cardiomyopathy, congestive heart failure, and ventricular arrhythmia), and neurological signs [96]. The unusual signs may relate to the accumulation of mutations in the SARS-CoV-2 genome, which led to changes in the virulence of the virus and result in unusual outcomes [97]. Therefore, further research is needed on SARS-CoV-2 mutations in humans and cats to increase awareness and suspicion in natural cases of SARS-CoV-2 infection, especially in asymptomatic cats.

### SARS-CoV-2 infections in dogs

Experimental studies in dogs found that SARS-CoV-2 replicated in the respiratory tract of dogs, but animals may not transmit the virus to other dogs [22,37]. Several inoculated dogs were positive for viral RNA, thus indicating the presence of viral replication, but dogs did not shed the infectious virus [22,37]. In addition, antibodies against SARS-CoV-2 were detected in inoculated dogs but were undetectable in naive co-housed dogs [22,37].

The natural infection of SARS-CoV-2 in dogs was reported in Hong Kong for the first time from a household infected with COVID-19. The dogs were found to be positive for viral RNA and seroconverted to SARS-CoV-2 [23]. Interestingly, the SARS-CoV-2 genomes from both dogs were identical to the viral genome from a related human case [23]. In addition, a serological study in dogs during the Wuhan outbreak showed that 1.69% of the dogs’ sera were positive for SARS-CoV-2 antibodies. The positive sera were collected from the owners with COVID-19, pet hospitals, and stray ani­mals [98]. The same result in Thailand showed that 1.66% of the sera collected from dogs during the outbreak were positive to SARS-CoV-2 antibodies [92].

In Italy, an epidemiological survey on SARS-CoV-2 infection in dogs reported that viral RNA was not detected, but several dogs with COVID-19 positive or negative owner found positive for SARS-CoV-2 neutralizing antibodies [28]. In France and Croatia, the seroprevalence of SARS-CoV-2 in dogs with COVID-19 positive owners was 15.4% [93] and 43.9% [99] respectively, whereas in the United Kingdom from the unknown owner status, the seroprevalence was 1.4% [100].

Several cases of SARS-CoV-2 infection in dogs were also reported in Rio de Janeiro, Brazil, from a household with a confirmed COVID-19 infection [55] and from a stray dog [54]. As many as 31% of dogs from households with patients with positive COVID-19 were positively infected with SARS-CoV-2, and some showed positive outcomes for antibodies to SARS-CoV-2 [55].

The first confirmed case of SARS-CoV-2 in a dog in the USA was announced on June 2, 2020. A German shepherd dog, which lived with another dog and the owner who was COVID-19 positive, developed the symptoms of respiratory illness and tested positive for viral RNA and neutralizing antibodies to SARS-CoV-2 [86,101]. In addition, several SARS-CoV-2 infection cases were reported by the OIE in follow-up reports with the numbers of 4, 8, 9, 10, 11, 12, 13, 14, 16, 17, 18, 20, and 23 [59-61,69-72,74,78-86,89,102-107]. In Texas was found that 1.7% of dogs from infected COVID-19 households were positive for the viral RNA, and 11.9% were positive for neutralizing antibodies to SARS-CoV-2 [94]. A serological study in Minnesota, USA, from April to June 2020 showed that 0.98% of dogs were seropositive for the N-protein SARS-CoV-2 [102].

The S-protein of SARS-CoV-2 interacted with the ACE2 of dogs. The analysis of canine ACE2 compared with human ACE2 contained five amino acid changes. These same amino acid changes also occurred in pig ACE2. These included the residues Gln24Leu, Asp30Glu, His34Tyr, Met82Thr, and Asp38Glu [95]. Changes in Gln24Leu and His34Tyr resulted in failure of hydrogen bond formation and in the weakening of the stability of the interaction between ACE2 and the S-protein of SARS-CoV-2 [103]. In contrast, the replacement of Asn90 residues with Asp resulted in a lack of N-glycosylation at position 90 [17,18,95]. *In silico* studies found the low susceptibility of dogs to SARS-CoV-2 infections [17,18,95]. In addition, no viral transmission was documented from inoculated animals to naive, close contact animals [22,37]. In the cases of natural infections, there was no confirmed evidence of COVID-19 transmission among dogs [23]. This suggests that dogs may be infected with SARS-CoV-2, but they have low susceptibility and have not transmitted the virus to other dogs [22,23].

## SARS-CoV-2 Infections in Wild Animals

### SARS-CoV-2 infections in big cats

Natural infections of SARS-CoV-2 in big cats have been reported in the tiger (*Panthera tigris*) [29,30,89,104-106], lion (*Panthera leo*) [29,30,104,105], snow leopard (*Panthera uncia*) [86,106], and cougar (*Puma concolor*) [61]. The first confirmed SARS-CoV-2 case was reported in the Bronx Zoo, New York City, USA, in tigers on April 4, 2020, and in lions on April 15, 2020 [104,105]. Tigers and lions showed clinical signs, such as dry cough and some wheezing, but no respiratory distress. All animals with clinical signs improved and recovered. The sources of infection were assumed to be transmissions from the zookeepers who had no clinical signs (asymptomatic) [104,105]. Epidemiologic and genomic data from the tiger and lion showed a different genotype of SARS-CoV-2, which indicated human-to-animal transmission from two different sources [29,30]. Furthermore, viral RNA shedding was found in feces and respiratory secretions of infected animals and persisted in the feces for more than 4 weeks [29,30]. Based on the infection timeline, it was assumed that the virus was transmitted from zookeepers to animals and subsequently to other animals in the same cage [29,30].

Another case in Tennessee, USA, found that three Malayan tigers (*P. tigris tigris*) exhibited clinical signs, including mild coughing, lethargy, and inappetence; all tigers were confirmed positive for SARS-CoV-2. It seems that the tigers were infected by the transmission of SARS-CoV-2 from an infected human. All tigers recovered [89,106]. In addition, other natural infection cases of SARS-CoV-2 in big cats and in the snow leopard at the Louisville Zoo, USA, were detected in December 2020 [61] and at the San Diego Zoo, USA, in July 2021 [60]; additionally, there was a cougar case in Texas, USA, in February 2021 [59]. In mid-September 2021, three tigers and six lions at the Smithsonian National Zoo, USA, were presumed positive for SARS-CoV-2 after they presented mild respiratory symptoms, such as coughing and sneezing, lethargy, and decreased appetite [107].

Natural cases of SARS-CoV-2 in Katanga lions (*P. leo bleyenberghi*) were reported in the Barcelona Zoo (Catalonia, Spain) from November to December 2020 [64]. These four lions had respiratory symptoms, such as sneezing, coughing, and nasal discharge, and developed antibodies against SARS-CoV-2 [64].

Recently, two Sumatran tigers (*P. tigris sumatrae*) at Ragunan Zoo, Jakarta, Indonesia, were confirmed positive for SARS-CoV-2 by RT-PCR, on July 15, 2021. These big cats presented with mild respiratory symptoms, such as lethargy, sneezing, shortness of breath, mucus secretion from the nose, and decreased appetite [65,66]. In India, nine lions [62] and three [63] Asiatic lions (*P. leo persica*) were reported to be positive to SARS-CoV-2 Delta variant in the B.1.617.2 lineage during May-June 2021 [62,63].

The susceptibility of the tiger, lion, leopard, and puma was analyzed by *in silico* studies by comparing the ACE2 of these animals with the human ACE2. ACE2 receptors from the tiger, cougar, and leopard (*Panthera pardus*) identified four amino acids changes, which were Gln24Leu, Asp30Glu, Asp38Glu, and Met82Thr and had His34 and N-glycosylated Asp90, the same as those for humans and cats [95,103,108]. By contrast, in lions, apart from having the same four amino differences as cats, a mutation of Asn90 to Asp resulted in the loss of N-glycosylation at site 90 [98]. Furthermore, a mutation was reported in His34 to Ser was also reported [95]. The His34 residue was considered a critical residue associated with the susceptibility of lions and tigers to SARS-CoV-2 infections [103]. The His34 to Ser mutation was predicted to decrease the binding stability between ACE2 and the SARS-CoV-2 S-protein [103]. This suggested that animals with His34Ser mutations had a lower susceptibility than animals with His34 [103].

Almost all animals had respiratory tract symptoms, with or without general symptoms of disease, such as lethargy or loss of appetite [29,30,59-61,65,66,89,104-106]. In addition, up to 96.5% of animals had a cough and 79% of animals had sneezing symptoms [96]. The appearance of the clinical signs may be explained by the ACE2 expressions in the ciliated bronchial epithelium cells from tigers and lions and in the endothelial blood vessels within the alveolar septa in tigers [109]. In view of the expressions of ACE2 in the respiratory tracts of big cats [109], the increasing number of natural infections of SARS-CoV-2 in these animals and the transmission of the virus from asymptomatic carriers [29,30,59-61,65,66,89,104-107], a SARS-CoV-2 vaccination program should be implemented in these big cats. There should be more concern about SARS-CoV-2 surveillance in wild animals to minimize the spread of SARS-CoV-2 within the animal population.

### SARS-CoV-2 infections in deer

The susceptibility of deer to the virus was investigated in studies *in vitro* and *in vivo*, as well as *in silico*. An *in vitro* study was performed in deer lung cells infected with SARS-CoV-2 isolate TGR/NY/20 [43] and human/USA/WA1/2020 [44]. It was found that SARS-CoV-2 replicated in white-tailed deer (*O. virginianus*) and mule deer (*Odocoileus hemionus*) lung cells [43,44], whereas the virus did not replicate in elk (*Cervus canadensis*) lungs cells [44].

Furthermore, in an *in vivo* study, SARS-CoV-2 was replicated in white-tailed deer fawns [43] and adult deer [44] and both groups of animals experienced subclinical viral infections [43,44]. Viral RNA was detected in nasal secretions and feces in fawns for longer periods than those in adult deer [43,44], in fawns during days 1-21 post-infection [43], and in adults during days 1-10 post-infection [44]. The virus replicated in the upper respiratory and gastrointestinal tracts and was shed from nasal, oral, and rectal swabs [44].

Viral transmission occurred from inoculated animals to indirect contact animals [43,44]. Viral RNA was detected in nasal, oral, or rectal swabs of co-housed animals [44]. Infectious viruses were detected in nasal secretions and the feces from indirect contact animals at days 2-7 post-infection [43]. Both inoculated and non-inoculated deer developed neutralizing antibodies [43]. Furthermore, despite the horizontal transmission between inoculated animals and indirect contact animals, the vertical transmission from the adult female deer to the fetus was also reported [44].

*In vitro* and *in vivo* studies showed a high susceptibility of deer to SARS-CoV-2 infections [43,44]. Recently, a serological survey during January-March 2021 in the USA (Michigan, Pennsylvania, Illinois, and New York states) has found SARS-CoV-2 antibodies in 40% of the wild white-tailed deer population [31]. In addition, antibodies against SARS-CoV-2 were detected in one and three sera samples in 2019 and 2020, respectively; however, these samples showed low percent inhibition values [31]. At present, the first confirmation of SARS-CoV-2 in the wild white-tailed deer was announced in Ohio, USA, on August 27, 2021 [110].

White-tailed deer, reindeer (*Rangifer tarandus*), and Père David’s deer (*Elaphurus davidianus*) were predicted to have a high susceptibility to SARS-CoV-2 infections [108]. Homology analyses of deer ACE2 revealed high similarities to humans ACE2 [108]. It showed four different amino acid residues (Asp30Glu, Leu79Met, Met82Thr, and Asn322His) and a Lys31Asn residue for Père David’s deer [108]. In addition, analyses of the interaction between ACE2 of these three species of deer and RBD of SARS-CoV-2 exhibited a high-binding score and indicated high susceptibility to viral infection [108]. Considering these *in silico* studies [108], the high susceptibility and transmissibility to SARS-CoV-2 infection [43,44], the high seroprevalence of SARS-CoV-2 in the wild white-tailed deer population [31], and the first confirmed SARS-CoV-2 infection case in wild white-tailed deer in the world, it is necessary to monitor the deer, its predators, and other wildlife populations [31].

## SARS-CoV-2 Infections in Farm Animals

### SARS-CoV-2 infections in cattle and sheep

In cattle (*B. taurus*), an *in vitro* study was performed in the bovine cell line, including turbinate, trachea normal, pulmonary artery, fetal bovine lung, and fetal bovine kidney cells. Cell lines were infected with SARS-CoV-2 isolate TGR/NY/20. This indicated that SARS-CoV-2 did not replicate [40]. However, another *ex vivo* study in organ cultures of respiratory tract cells demonstrated that SARS-CoV-2 replicated in lung and trachea cells. The respiratory tract was also shown immunoreactive to the polyclonal antibody of ACE2 [41].

An *in vivo* study of SARS-CoV-2 infection in cattle showed that the virus replicated but was not transmitted [40,41]. Six-week-old calves exhibited mild symptoms, such as a high temperature and mild cough. The virus replicated, but viral shedding was not found. The calves developed neutralizing antibodies against SARS-CoV-2, but this antibody titer did not persist for more than 21 days [40]. Another study in older calves revealed that the virus replicated, but the calves did not shed the virus and there were no clinical signs [42].

Homogenetic analyses of ACE2 of the family Bovidae, including cattle (*B. taurus*), water buffalo (*Bubalus bubalis*), wild goat (*Capra aegagrus*), goat (*Capra hircus*), and sheep (*O. aries*), with human ACE2 exhibited high similarity. This analysis identified four amino acid residues different from those of human ACE2: Asp30Glu, Leu79Met, Met82Thr, and Asn322Tyr. Furthermore, the evaluation of the binding contact between ACE2 of those animals with RBD in the S-protein of SARS-CoV-2 predicted medium susceptibility to SARS-CoV-2 infection, at the same level as documented in the cat [108]. In addition, ACE2 receptors were expressed in the bronchiole epithelia of cattle and sheep but not in the nasal mucosa and alveoli [109]. By contrast, ACE2 receptors in cats were expressed in alveoli and Type I pneumocytes [109]. However, an *in vivo* study found that the infectious virus was not detected in cattle. This may indicate that cattle had low susceptibility to SARS-CoV-2 infections [40,42].

The susceptibility of sheep to SARS-CoV-2 infection was investigated in *ex vivo* organ cultures of respiratory tract cells infected with SARS-CoV-2 with D614 and SARS-CoV-2 with D614G. The results demonstrated that sheep lung and trachea cells exhibited ACE2 receptors and thus supported the replication of both SARS-CoV-2 variants [41]. This indicates that SARS-CoV-2 can infect sheep, but further *in vivo* studies are needed to confirm the susceptibility of sheep to SARS-CoV-2 infection. Likewise, research on the susceptibility of other ruminant groups to SARS-CoV-2 infections still requires further *in vitro* and *in vivo* research studies.

### SARS-CoV-2 infections in pigs

The susceptibility of pigs to SARS-CoV-2 infections was investigated *in vitro* using swine cell lines. Swine testicular and kidney cells (SK-6 and PK-15) [45,46] supported SARS-CoV-2 replication. In contrast, SARS-CoV-2 did not replicate in *ex vivo* respiratory organ cultures from pigs [41].

*In vivo* studies in domesticated pigs (*S. scrofa domesticus*) found no viral replication and transmission of SARS-CoV-2 from inoculated animals to contact-naive animals [22,45-47]. Viral RNA was not detectable in oropharyngeal and rectal swabs from pigs inoculated with 10^5^ PFU of CTan-H or naive animals at all-time points, and there were no antibodies to SARS-CoV-2 [22]. Pigs infected with 10^5^ TCID_50_ of 2019_nCoV Muc-IMB-1 yielded the same results [45]. Inoculated and naive-contact animals had no clinical signs. Viral RNA, antibodies, and organ lesions after necropsy were also not detected [45]. Both those studies challenged pigs intra-nasally [22,45]. Another study that carried out the challenge through the intranasal, oral, and intratracheal routes simultaneously obtained the same results, despite the higher dose (dose 10^6^ TCID_50_ of SARS-CoV-2) [46]. Meanwhile, pigs inoculated with 10^5.8^ TCID_50_ of SARS-CoV-2 intravenously and intramuscularly were shown to have low levels of anti-SARS-CoV-2 antibodies, despite the fact that they did not show clinical signs, and viral RNA was not detected in nasal or rectal swabs [47].

Although the previous studies that challenged pigs with SARS-CoV-2 through intranasal, intratracheal, oral, intramuscular, and intravenous routes showed that pigs were not susceptible to SARS-CoV-2 infections [22,45-47], there were two research groups reported different results [48,49]. First, pigs aged 8 weeks were challenged with 10^6^ PFU/animal of SARS-CoV-2 isolate hCoV-19/Canada/ON-VIDO-01/2020 via the nasal and pharynx routes. It was the first study that detected low-level viral RNA in nasal washing and oral fluids after inoculation. However, it was not detectable in other swab samples (oral, nasal, and rectal swabs). The study found neutralizing antibodies against SARS-CoV-2 at low levels in two pigs. One pig presented cough and mild depression symptoms from day 1 to 4 post-infection. The infectious virus was detected in this pig in the submandibular lymph node at day 13 post-infection [48]. A second study on pigs involved infections with 6.8×10^6^ TCID_50_ of the SARS-CoV-2 isolate TGR/NY/20 through the intratracheal, intranasal, and intravenous routes. Viral RNA in nasal/oral and rectal swabs and neutralizing antibodies against SARS-CoV-2 from all groups of administration routes were detectable but transient. Furthermore, some tissues (tonsils, mandibular lymph node, and tracheobronchial lymph node) from inoculated animals showed weak positivity for viral RNA, but the infectious viruses were not isolated successfully. That study proved that inoculation of the virus through these routes could not produce the infectious virus, and there were no viral transmissions from inoculated animals to naive-contact animals [49].

Several studies predicted the susceptibility of pigs to SARS-CoV-2 infections based on comparisons of pig ACE2 with human ACE2 [95,108]. These studies found five amino acid changes in pig ACE2, as in dogs [95,108]. There are mutations of Gln24Leu, Asp30Glu, and Met82Thr in pigs and dogs [95,108], His34 to Leu in pigs and Tyr in dogs, and Asn90 to Thr in pigs and Asp in dogs [95,108]. Mutations of Gln24 to Leu and His34 to Leu or Tyr led to the failure of hydrogen bond formation between the SARS-CoV-2 S-protein and porcine ACE2 receptors [95,108]. In addition, mutations of Asn90 to Thr or Asp led to a lack of glycosylation site at position 90 [95,108]. Based on these *in silico* studies, pigs and dogs exhibited low susceptibility to SARS-CoV-2 [95,108], but dogs have been shown infected with SARS-CoV-2 naturally [24,54,55,86,93,101].

*Ex vivo* [41] and *in vivo* studies [22,45-47] in swine respiratory tract cells found no SARS-CoV-2 replication. On the contrary, infection with higher doses showed weak positive viral RNA in swabs [48,49], and SARS-CoV-2 RNA and protein of inoculated animals were undetectable in respiratory tract cells [41,46,48]. The distribution of ACE2 protein on the tissues showed no expression in the upper and lower respiratory tract cells [41,109], but the mRNA type was found to be weakly expressed [49]. However, it was overexpressed in the small intestine [109] and kidney [41,49]. This may explain the fact that SARS-CoV-2 replicated in kidney cells [45,46] but not in the respiratory tract cells of pigs [41,45,46,49]. Those experimental studies were consistent with *in silico* predictions and indicated that pigs have a low susceptibility to SARS-CoV-2 infections [108].

### SARS-CoV-2 infections in minks

The first case of natural infection of SARS-CoV-2 in minks (*Neovison vison*) was reported in two farms in the Netherlands in April 2020 [34]. These animals revealed severe respiratory diseases and increased mortality. The clinical signs included breathing difficulties and nasal exudate. SARS-CoV-2 viral RNA and viral antigen were detected in the upper and lower respiratory tracts [34]. Histopathological features included the thickening and degeneration of alveolar septa, which indicated acute severe interstitial pneumonia or diffuse alveolar damage [34,56]. Before the SARS-CoV-2 outbreak occurred in the mink farm, a worker in the farm tested positive for SARS-CoV-2, indicating the probable transmission from the human to mink [34].

In addition, SARS-CoV-2 infected minks were reported in Denmark around June 2020 [111]. Similar findings were reported in several countries in Europe, which included Spain in July 2020 [112,113], Italy in August 2020 [112,113], Sweden in October 2020, Greece, France, Poland, and Lithuania in November 2020, the second infection in a mink farm in Poland on 30 January 2021, and in Latvia in April 2021 [58,112,113]. In the Netherlands and Denmark, the virus spread rapidly among minks, resulting in respiratory diseases and increased mortality [35,111].

The first case was reported in August 2020 in two commercial mink farms in the USA. The clinical findings included respiratory signs and sudden death. It was assumed that a mink was infected from SARS-CoV-2 infected people who contacted the mink and the virus spread it among minks in these farms [87]. A total of 177,357 suspected minks and the deaths of 16,130 minks due to SARS-CoV-2 infections were reported in mink farms in Utah, Michigan, Wisconsin, and Oregon, USA, from June to October 2020, as OIE reported in the follow-up reports No. 15, 16, 19, 20, 21, 22, 25, and 26 [71-73,82,87-90].

The SARS-CoV-2 genome in the mink farm in the Netherlands had a high diversity [35]. There were five clusters, among which three clusters (A, C, and E) contained the mutation of aspartate 614 to glycine (D614G) that was found in general human populations and in cases related to minks [35]. In Denmark, mutations that occurred in the ORF1b gene were mutations of threonine 730 to isoleucine (T730I) and proline 314 to leucine (P314L). In contrast, in the ORF3a gene, there was a mutation of histidine 182 to tyrosine (H182Y). Finally, in the nucleoprotein gene, there were mutations of arginine 203 to lysine and glycine 204 to arginine [111]. In addition, D614G and Y453F mutations occurred in the spike gene [111]. The SARS-CoV-2 variant T730I was found in humans and in the mink population in Jutland, Denmark, and in human from New Zealand [111]. A H182Y mutation within ORF3a appeared in all minks in Denmark and in human cases related to the mink. Even if it was a rare mutation, it was also found in a mink farm in the Netherlands [111]. Recently, the new variant of SARS-CoV-2 that contained the deletions of histidine 69 (H69) and valine 70 (V70) has been reported. Some mutations developed in mink farms and in 12 humans with COVID-19 who lived around the mink farms in Jutland included Y453F, D614G, isoleucine 692 to valine (I692V), and methionine 1229 to isoleucine (M1229I) [57]. The deletion of H69 and V70 within the spike gene occurred in mink farms probably as an adaptation of the virus to increase its binding ability to the receptor [114]. The same finding was revealed in Poland [115]. Mutations occurred in the spike gene, which resulted in alterations of the amino acids glycine 75 to valine, methionine 177 to threonine, cysteine 1247 to phenylalanine, and contained the amino acid mutation Y453F [115], as previously reported in the mink farm in Denmark [57,111].

D614G and Y453F are two interesting mutations in the S-protein of SARS-CoV-2. These are specific mutations found in the mink and are related to the mutations found in humans on the mink farm [35,111]. Mutations of D614G in S-protein were found predominantly in the human population, in the mink farm in Denmark and the Netherlands [35,111]. Furthermore, Y453F mutation was found in mink farms in the Netherlands and was related to human cases in mink farms in Denmark [111]. The change of aspartate residue at position site 614 to glycine and the tyrosine residue at position site 453 to phenylalanine were a form of virus adaptation to allow the virus to enter host cells; this efficiently increased ACE2 binding in minks and humans [116]. In addition, the mutation of Y453F reduced the efficiency of antibody therapy and convalescent serum/plasma therapy from patients with COVID-19, thus reducing the success of therapy and increasing the risk of death in patients [116].

The SARS-CoV-2 genome obtained from the mink samples was highly similar to humans associated with mink farms in the Netherlands and Denmark [35,111], indicating viral transmissions from the mink workers to the animals [35]. Subsequently, the spreading of the virus among minks in the farms occurred by inhalation of spray droplets from sneezing and coughing or inhalation of aerosol microparticles (<5 mm) that contained infectious viruses [117,118]. This has been proven by finding viral RNA in dust samples collected using stationary air sampling (over 5-6 h periods) in the mink farm during the outbreak [34]. Furthermore, based on genomic and epidemiological studies, it appeared that SARS-CoV-2 was transmitted from humans to minks and spread among minks following the appearance of several new mutations; it was then transmitted back to humans, as was also observed in the Netherlands and Denmark [35,111], making it possible to transfer the virus to other sites [112].

The spread of SARS-CoV-2 from the mink to the surrounding environment or to other animals that live at the farms is also possible [112,119]. This is based on the finding of viral RNA in airborne dust collected at locations 2-3 m from farms, in fur and straw from infected farms, and in the feet of seagulls that often forage on mink farms in Denmark, thus making it possible to transfer the virus to other sites [112]. The dogs and cats on the farm were also positive for viral RNA, and some dogs and cats had antibodies to SARS-CoV-2 [112]. A study from the Netherlands [119] reported that viral RNA was identified in stray cats that lived near farm sites and cats and dogs that lived on the farm [119]. The authors presumed that the stray cats were infected by the minks, but the source of viral infections in dogs has not been determined [119].

SARS-CoV-2 transmission from humans to minks, minks to minks, and minks to humans or other animals was found [35,111,112,119]. In addition, indirect transmission through dust or objects around the mink farm contains the active virus [58,119]. There was evidence of the possibility of the emergence of new strains because of new mutations or accumulations of mutations in the viral genome in the mink group, which were faster and more virulent [57,111,115,116]. Hence, it is necessary to consider mitigation strategies to manage outbreaks in animals, humans globally, especially those related to transmission cases among animals, from animals to humans, and humans to animals. It is also crucial to protect stray animals and wild animals around mink farms.

### SARS-CoV-2 infections in poultries

To evaluate susceptibility of poultries to SARS-CoV-2 infection, several experimental studies have been conducted, including in chickens (*Gallus gallus domesticus*), turkeys (*Meleagris gallopavo*), pekin ducks (*Anas platyrhinchos domesticus*), Japanese quails (*Coturnix japonica*), and in white Chinese geese (*Anser cygnoides*) [22,45,50]. These domesticated fowl were infected intra-nasally or ­oculo-oronasally and later introduced to naive animals. All studies reported that viral RNA was not detected in any oropharyngeal and cloacal swabs collected from inoculated animals or naive animals. In addition, all these birds were seronegative for SARS-CoV-2 [22,45,50]. All animals showed no clinical signs during the study, and any lesion was detected at necropsy [45,50]. Similarly, embryonated chicken eggs (ECEs) were usually used for isolation, and the laboratory host system in the vaccine production exhibited no viral replication in ECEs [45,50]. All these studies on poultry and ECEs showed that the viral RNA cannot be replicated and transmitted among birds [22,45,50].

Despite experimental studies, it was found that chickens that had indirect contact with the mink farm outbreak were negative for SARS-CoV-2 viral RNA [112,119]. It was also reported that wild birds trapped in the mink farms affected, including hundreds of seagulls with other birds, including one hooded crow (*Corvus cornix*), a jackdaw (*Corvus monedula*), and a common kestrel (*Falco tinnunculus*), were found negative for SARS-CoV-2 RNA [112]. This was in accordance with the predictions of *in silico* studies [95]. The class Aves, including chickens and ducks, had ACE2 receptors that did not match the S-protein of SARV-CoV-2 [95]. Analyses conducted to compare the chicken and duck ACE2 receptors with human ACE2 receptors showed that the receptors of these avian species contained ten amino acids changes and lacked the N-glycosylation at position site 90 [95]. These changes affected the amino acid residue involved in the binding of ACE2 to the SARS-CoV-2 S-protein, in chicken including Gln24Glu, His34Val, Leu79Asn and Met82Arg, and Gly354Asn, and in ducks was His34Val, Leu79Asn, Met82Asn, and Gly354Asn [95]. This change also occurred in Tyr83Phe, which resulted in the failure of hydrogen bond formation, and in Asp30Ala, which resulted in the lack of salt bridge formation [95]. Therefore, these findings may explain the inability of ACE2 receptors in the bird group to bind to the S-protein of the SARS-CoV-2. These findings suggest that poultry are not susceptible to SARS-CoV-2 infections [22,45,50].

### SARS-CoV-2 infections in other animals

SARS-CoV-2 infection has been reported in several animals. Gorillas (*Gorilla gorilla*) at the San Diego Zoo, USA, were found positive for SARS-CoV-2 on January 11, 2021. Despite appearing to have a mild cough, stuffy nose, and lethargy symptoms, they recovered [91]. Confirmation of COVID-19 was reported in Asian small-clawed otters (*Aonyx cinereus*) in Georgia, USA, in April 2021 [120]. These otters, which includes in the family Mustelidae that the same family with minks, showed clinical signs, such as sneezing, runny noses, mild lethargy, and coughing [120]. Recently, several animals have been reported to be infected with SARS-CoV-2, including animals at a zoo in Illinois, USA, that was a binturong (*Arctictis* binturong) and a fishing cat (*Prionailurus viverrinus*) on October 5, 2021, [121] and a South American coati (or coatimundi, *Nasua nasua*) on October 14, 2021 [122]. Furthermore, two hyenas at Denver Zoo in Colorado, USA [123] were tested positive for SARS-CoV-2 with other animals in the zoo, including lions and tigers, on November 5, 2021 [123]. The two hippos at a zoo in Antwerp, Belgium were positive for SARS-CoV-2 infections on December 6, 2021 [124].

Animals from infected mink farms, such as chickens, rabbits, and horses, tested negative for SARS-CoV-2 [112]. PCR-negative outcomes for SARS-CoV-2 were also found in a group of wild animals collected in the areas around the infected mink farms from October to November 2020 in Denmark, including red foxes (*Vulpes vulpes*), badgers (*Meles meles*), least weasel (*Mustela nivalis*), polecats (*Mustela putorius*), otter (*Lutra lutra*), beech martens (*Martes foina*), and raccoon dogs (*Nyctereutes procyonoides*), as well as in feral mink (*N. vison*) [112]. SARS-CoV-2 infections have not been reported in other wild animals, pets, and farm animals that have close contact with humans, such as horses, goats, camels, and buffaloes, have not been reported. This requires further investigation in terms of both the detection of viral RNA and serological surveys.

Recently, there have been many reported cases of COVID-19 in animals. To prevent SARS-CoV-2 infections in various animals, both pets and wild and farm animals, vaccines have been developed, including a vaccine from Zoetis company, Carnivac-Cov, and the LinearDNA™ COVID-19 vaccine [125-127]. Zoetis has developed a subunit recombinant vaccine for the SARS-CoV-2 S-protein for wild animals. It has been used to vaccine some species of wild animals in several zoos and sanctuaries in the USA and Canada, including orangutans, bonobos, hyenas, chimpanzees, and lions [125,126]. Thus, Russia has developed Carnivac-Cov, an inactivated vaccine, and has been on clinical trials in dogs, cats, foxes, and minks [125]. The Linear DNA™ COVID-19 vaccine has been developed by Applied DNA Sciences (USA) and EvviVax (Italy) for use in domestic felines [127]. The safety and immunogenicity of this vaccine in cats showed to be well tolerated and induced high titers of SARS-CoV-2 neutralizing antibodies [127], while the safety and immunogenicity in minks are currently in progress of research [128]. Furthermore, successful immunization of animals could protect animals from SARS-CoV-2 infections and prevent virus transmission among animals and cross-species. Therefore, it reduces the risk of the emergence of new mutations of SARS-CoV-2 [125,129].

## Conclusion

The susceptibility of animals to SARS-CoV-2 is very different depending on the family. Felines, including domestic cats and big cats, are susceptible species where virus transmission between animals has also been detected. Other wild animals that were found to be infected as natural infections in the zoos were gorillas, otters, a binturong, a fishing cat, a coatimundi, hyenas, and hippos. Livestock, such as cattle, sheep, and pigs, have a low susceptibility to SARS-CoV-2 infections, whereas poultries have been shown to be less susceptible to SARS-CoV-2 infection.

In most cases, infection of SARS-CoV-2 in animals was through close contact with humans, including in domesticated animals, big cats, and other wild animals in zoos. This also occurred in white-tailed deer and minks. In white-tailed deer, the virus can transmit to other deer that are in close contact or to its fetus experimentally. Furthermore, it is suspected that SARS-CoV-2 may have spread to the white-tailed deer population naturally with the finding that the seroprevalence of SARS-CoV-2 in the deer population was quite high. In minks, the virus infections were being transmitted from humans and be spread among minks and then undergone adaptation and spread back to humans. Presumably, the virus in minks and white-tailed deer were also possible to be transmitted to other animals because of the large number of infected animals and the high seroprevalence rate in these two animal species.

When infecting humans or animals, viruses generate several mutations and accumulate; then the mutation will be transmitted to other humans or animals. Some mutations increase the level of viral virulence, and some cause resistance to antibodies or convalescent plasma therapy. Therefore, it is necessary to increase the awareness of rapidly mutating viruses and prepare various forms of appropriate therapies and treatments. Not only do vaccines need to be developed, but also research related to the development of antivirals and therapeutic management, as well as comprehensive strategies for mitigating infectious and dangerous diseases are also necessary. This knowledge may contribute to the management of the SARS-CoV-2 pandemic in humans and animals.

## Authors’ Contributions

GM: Conception of idea and drafted and revised the manuscript. GM, AR, and RI: Literature search. AR and RI: Editing of the manuscript. IR and IdB: Conception of idea, literature search, and reviewing the manuscript. All authors read and approved the final manuscript.
